# Ozone therapy as an alternative method for the treatment of diabetic foot ulcer: a case report

**DOI:** 10.1186/s13256-021-02829-y

**Published:** 2021-05-13

**Authors:** Navid Faraji, Rasoul Goli, Babak Choobianzali, Soheyla Bahrami, Ali Sadeghian, Nazila Sepehrnia, Mahmoodreza Ghalandari

**Affiliations:** 1 Department of Medical-Surgical Nursing, Nursing and Midwifery Faculty, School of Nursing and Midwifery, Urmia University of Medical Sciences, Campus Nazlu, 11 KM Road Seru, 575611-5111 Urmia, West Azerbaijan Iran; 2 Department of Emergency Medicine, Medicine Faculty, Urmia University of Medical Sciences, Campus Nazlu, 11 KM Road Seru, 575611-5111 Urmia, West Azerbaijan Iran; 3 Department of Medical-Surgical Nursing, Nursing and Midwifery Faculty, School of Nursing and Midwifery, Islamic Azad University of BonaB Branch, Velayat Highway, 555178-5176 Bonab, East Azerbaijan Iran; 4 Department of Medical-Surgical Nursing, School of Nursing and Midwifery, Zanjan University of Medical Sciences, Dr.Sobouti Blvd., 451395-6111 Zanjan, Iran; 5 Department of Psychiatric Nursing, Nursing and Midwifery Faculty, School of Nursing and Midwifery, Tabriz University of Medical Sciences, Shahnaz Street, 575611-5111 Tabriz, East Azerbaijan Iran

**Keywords:** Diabetic foot, Ozone, Silver, Wound, Infection

## Abstract

**Background:**

Diabetic foot ulcer (DFU) is one of the most important complications of diabetes that can lead to amputation. Treatment of DFUs is a major challenge and places a heavy economic and social burden on patients and their families.

**Case presentation:**

The present case report is of a 52-year-old kurdish male patient with a 7-year history of type 2 diabetes. While on a bike ride, he sustained a traumatic injury to his right leg, which caused a deep gash measuring 14 × 5 cm on the tibia. During the hospital stay, no improvement was observed after routine wound care including suturing, antibiotic therapy, and dressing change. The patient was referred to our wound-care team. In the first step, the necrotic tissues of his foot ulcer were irrigated and then debrided using mechanical debridement and saline. Next, the patient underwent a 70 μg/dL dose of ozone therapy over a 30-day period in 10 sessions (one 20-minute session every 3 days). Between each session, the patient's wound was wrapped in silver-containing gauze bandages. After 1 month of wound-care using ozone therapy, the patient's foot ulcer had healed and he was discharged from our wound-care service with a stable and good general condition.

**Conclusion:**

Considering the effectiveness of ozone therapy along with silver-containing dressing in the treatment of DFUs, wound-care teams can utilize it as an adjunct to the standard methods of DFU treatment.

## Background

Diabetes is a metabolic disorder characterized by hyperglycemia, which results from a decrease in insulin secretion, a decrease in the cells' response to insulin, or both [1]. As the prevalence of diabetes rises, its secondary side effects increase, among which diabetic foot ulcer (DFU) is the most important [2]. Diabetic neuropathy and peripheral vascular disease (PVD) are the two main causes of DFU [3]. DFU is the most common cause of hospitalization in diabetic patients, among which about 25% are at risk of developing DFU [4]. Moreover, 20% of DFUs culminate in amputation [5], and treatment of DFUs imposes a great economic and social burden on the health care system and patients' families. Therefore, special attention is needed regarding the management of DFUs [4].

Common treatments for DFUs include glycemic control, wound debridement, vascular surgery, antibiotic therapy (topical and systemic), pressure offloading, and wound dressings such as silver, hydrogel, alginate, hydrocolloids, and foam dressings. Other developed treatment approaches include growth factor therapy, skin replacement [1], maggot therapy, ozone therapy, stem cell therapy [2], hyperbaric oxygen therapy [3], and therapeutic application of extracellular matrix proteins [2, 4, 5].

Because silver ions possess antibacterial and disinfectant properties, silver-containing dressings are widely used to treat DFUs. Generally, silver dressings are used to treat local wound infections, but they can also be used in combination with systemic antibiotics to treat systemic wound infections [6].

Ozone therapy refers to the use of ozone gas for treating a disease or wound, especially DFUs [2]. Ozone is a gas comprising three oxygen atoms that are rapidly broken down. This gas increases the cell membrane permeability to glucose, improves metabolism of oxygen, promotes oxidative preconditioning [5], and stimulates the endogenous antioxidant system, all of which can ultimately lead to the prevention of cell neuropathy and improvement in tissue perfusion and oxygenation. Ozone also has anti-inflammatory and antibacterial effects [7–9].

Although several studies have shown the effectiveness of ozone therapy in treating infections and wounds because of its antimicrobial properties [2, 8], the extensive use of this method as a treatment is restricted due to its side effects, including toxic effects on the respiratory tract [6]. It should be noted that these side effects are dose-dependent, and application requires a closed system ensuring no escape of ozone [10]. Ozone therapy is a controversial approach, and additional evidence is needed to support its application. External wounds, especially in the extremities such as DFUs, are eminently suitable and practical sites for ozone therapy because they can be easily treated using an appropriate dose of ozone in the form of a transcutaneous ozone gas bath, which can almost guarantee no escape of ozone into the surrounding air. This case report describes the treatment of a DFU using ozone therapy along with a silver dressing in a patient who showed dramatic post-traumatic wound improvement.

## Case presentation

**Fig. 1:**
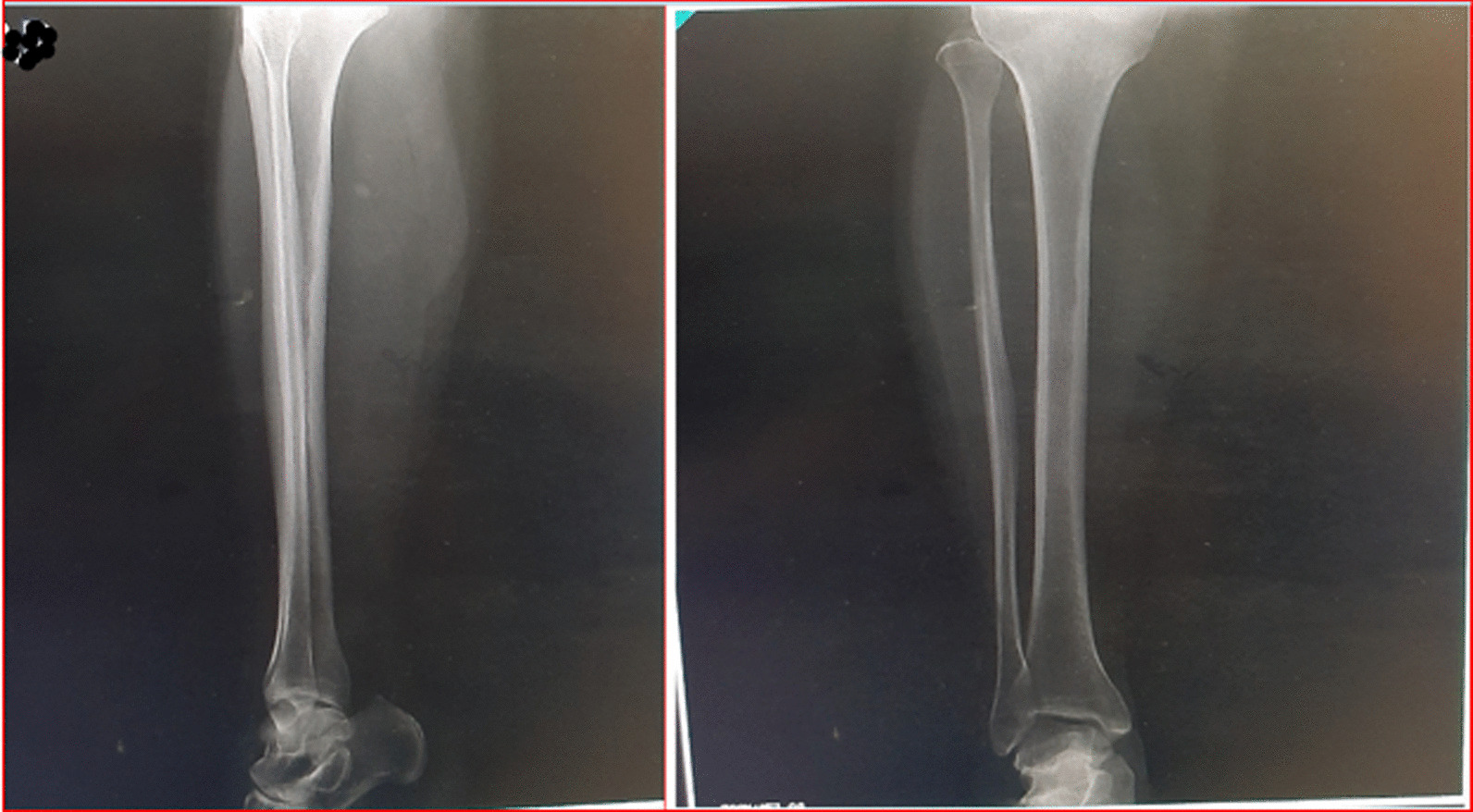
Anterior/posterior and lateral radiographs of the right tibia bone

**Fig. 2:**
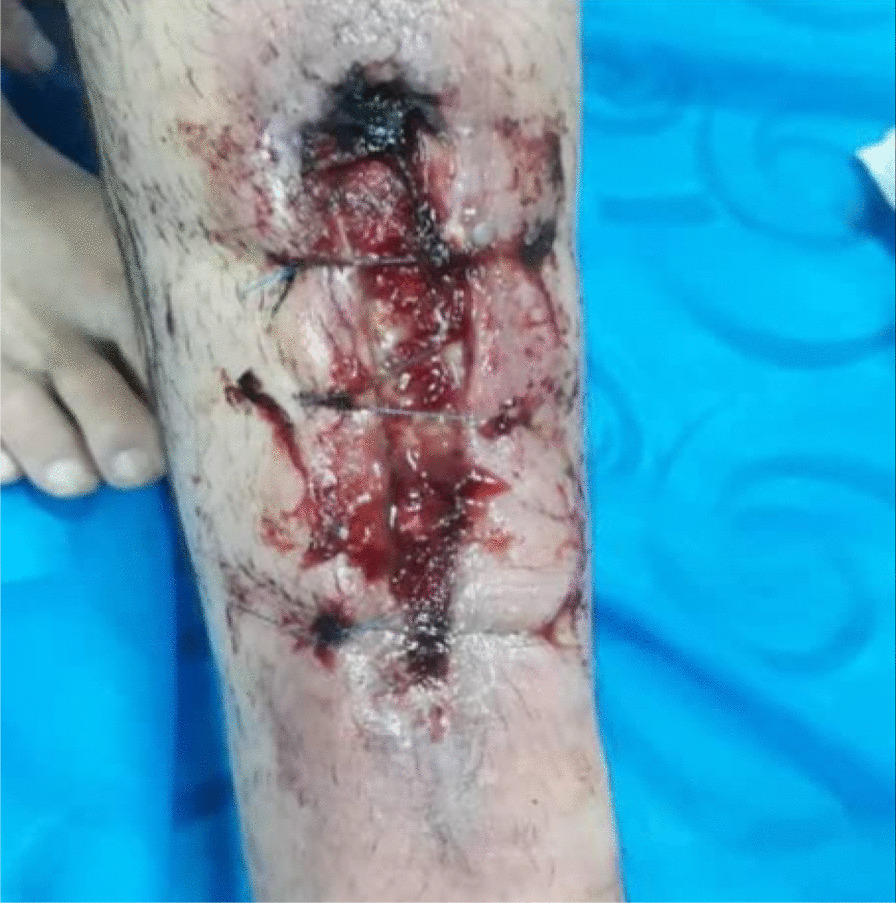
Stitched diabetic foot ulcer after discharge from hospital

This case report is of a 52-year-old kurdish male patient with a 7-year history of type 2 diabetes. He was from a village located in West Azerbaijan (a province in northwestern Iran) and had a primary education. About 6 months earlier, on 13 April 2020, he sustained a traumatic injury to the right tibia while on a bike ride. He was taken to the emergency department of Imam Khomeini hospital, Urmia and was hospitalized for a week. The wound was in the form of a deep skin gash measuring 14 × 5 cm on the right tibia without any evidence of fracture in the anteroposterior and lateral radiographs of the right leg (Fig. 1). During the hospital stay, the patient's wound was sutured using 3-0 nylon sutures (Fig. 2) and treated using intravenously administered antibiotics including cefazolin 1 g every 6 hours (three times daily), ciprofloxacin 400 mg every 12 hours (twice daily), and clindamycin 600 mg every 8 hours (three times a day). His foot ulcer was dressed twice a day using saline wound dressing. The patient's vital signs on admission to the hospital were as follows: temperature 37.6 °C, respiration rate 17 breaths per minute, pulse rate 96 beats per minute, blood pressure 130/85 mmHg, and oxygen saturation 93%.

The patient's laboratory data are shown in Table 1.

**Table 1:** The patient's laboratory data on first admission

*Urinalysis*	*Complete blood count*	*Biochemistry*
Color: yellow	White blood cells: 23,000 μL	Blood urea nitrogen: 14.8 mg/dL
Appearance: semi-clear	Red blood cells: 5,490,000 μL	Creatinine: 1.1 mg/dL
pH: 5	Hemoglobin: 12.5 g/dL	Urea: 23.2 mg/dL
Specific gravity: 1.015	Hematocrit: 39%	Calcium: 8.75 mg/dL
Protein: negative	Mean corpuscular volume: 64.9 fL	Phosphorous: 4.5 mg/dL
Sugar: negative	Mean corpuscular hemoglobin: 19.5 pg	Sodium: 146 mEq/dL
Blood: negative	Mean corpuscular hemoglobin concentration: 30.46 g/dL	Potassium: 3.9 mEq/dL
Urobilinogen: negative	Red cell distribution width–CV: 15.7%	Aspartate transaminase: 62 U/L
Ketone: negative	Red cell distribution width–SD: 42.3 fL	Alanine transaminase: 80 U/L
Nitrite: negative	Platelets: 249,000 μL	Alkaline phosphatase: 450 U/L
Bilirubin: negative	Platelet distribution width: 16.0	Bilirubin total: 0.9 mg/dL
White blood cells: 4–5/HPF	Mean platelet volume: 8 fL	Bilirubin direct: 0.4 mg/dL
Red blood cells: 0–2/HPF	Procalcitonin: 0.199%	Blood sugar: 345 mg/dL
Epithelial cells: 3–5/HPF	*Serology*	Low-density lipoprotein: 89 mg/dL
Mucus: not seen	C-reactive protein: positive (+2)	High-density lipoprotein: 37 mg/dL
Casts: not seen	*Thyroid function*	Cholesterol: 195 mg/dL
Bacteria: few	Thyroid-stimulating hormone: 12 mIU/L	Triglycerides: 130 mg/dL
Crystal: not seen	Free thyroxine: 10 pmol/L	Hemoglobin A1c: 7.0%
Yeast: not seen	Free triiodothyronine: 3.5 pmol/L	

*HPF* high-power field, *CV* coefficient of variation, *SD* standard deviation

During history-taking and physical examination, the patient mentioned a history of beta thalassemia trait, hyperthyroidism, and benign prostatic hyperplasia, for which he had undergone transurethral resection of the prostate (TURP) 3 years earlier. No pathological findings were noted during the neurological examination, which included an assessment of motor and sensory systems, gait and stance, coordination, mental status, reflexes, and nerve functioning. Moreover, during the history-taking, it was found that he was not taking his medication regularly and was not following a sensible diet. His blood glucose was also not in a normal range. The patient also had a family history of diabetes mellitus, hypertension, and beta thalassemia trait. He was a smoker (23 pack-years), but he denied any drug or alcohol addiction. He was also from a low-income family and commanded the full social support of his family. He was a farmer and had a small farm for keeping livestock. He was treated with metformin hydrochloride 500 mg tablets twice a day after meals and glibenclamide 2.5 mg tablets twice a day, 30 minutes before breakfast and dinner, to control his blood sugar level. He also took levothyroxine sodium 0.1 mg tablet once daily, 1–1.5 hours before breakfast.

**Fig. 3:**
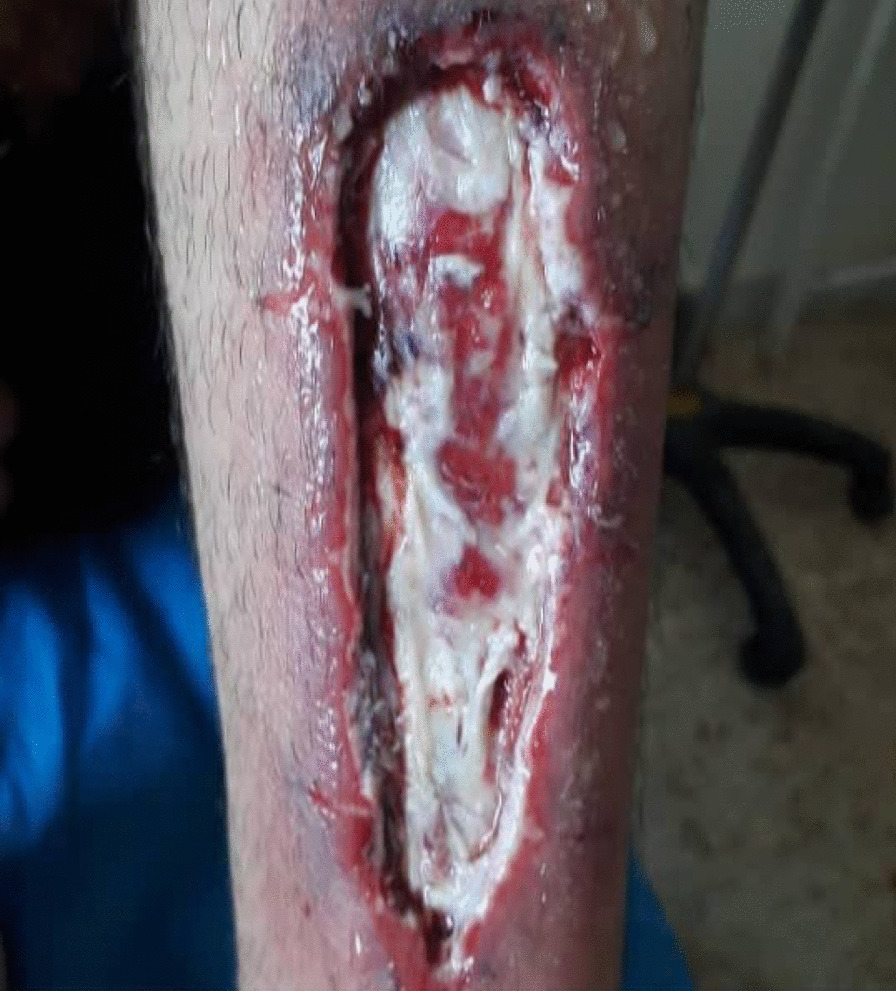
Diabetic foot ulcer after removing the stitches

After 1 week of hospital stay, the patient was discharged with cephalexin 500 mg capsules. He was also ordered to do daily saline wound dressing. About 10 days after hospital discharge, the wound sutures were removed. However, the patient complained that his wound did not heal as it should and the wound area was giving off an unpleasant odor. On 3 May 2020, he was referred to our wound-care team, since no improvement was noted in the healing of his DFU using conventional treatment (Fig. 3).

The patient's vital signs upon the second admission were as follows: temperature 37.1 °C, respiratory rate 16 breaths per minute, pulse rate 87 beats per minute, blood pressure 125/80 mmHg, and oxygen saturation 93%.

The patient's laboratory data during the second admission are shown in Table 2:

**Table 2:** The patient's laboratory data in our wound-care center (second admission)

*Urinalysis*	*Complete blood count*	*Biochemistry*
Color: yellow	White blood cells: 13,000 μL	Blood urea nitrogen: 15.1 mg/dL
Appearance: semi-clear	Red blood cells: 5,560,000 μL	Creatinine: 1.3 mg/dL
pH: 5	Hemoglobin: 12.9 g/dL	Urea: 24.1 mg/dL
Specific gravity: 1.013	Hematocrit: 42%	Calcium: 8.73 mg/dL
Protein: negative	Mean corpuscular volume: 64.5 fL	Phosphorous: 4.5 mg/dL
Sugar: negative	Mean corpuscular hemoglobin: 21.43 pg	Sodium: 145 mEq/dL
Blood: negative	Mean corpuscular hemoglobin concentration: 32.44 g/dL	Potassium: 3.8 mEq/dL
Urobilinogen: negative	Red cell distribution width–CV: 15.6%	Aspartate transaminase: 65 U/L
Ketone: negative	Red cell distribution width–SD: 42.1 fL	Alanine transaminase: 83 U/L
Nitrite: negative	Platelets: 248,000 μL	Alkaline phosphatase: 421 U/L
Bilirubin: negative	Platelet distribution width: 16.0	Bilirubin total: 0.9 mg/dL
White blood cells: 3–5/HPF	Mean platelet volume: 8 fL	Bilirubin direct: 0.5 mg/dL
Red blood cells: 0–2/HPF	Procalcitonin: 0.198%	Blood sugar: 286 mg/dL
Epithelial cells: 3–6/HPF	*Serology*	Low-density lipoprotein: 89 mg/dL
Mucus: not seen	C-reactive protein: positive (+1)	High-density lipoprotein: 36 mg/dL
Casts: not seen	*Thyroid function*	Cholesterol: 183 mg/dL
Bacteria: few	Thyroid-stimulating hormone: 11 mIU/L	Triglycerides: 124 mg/dL
Crystal: not seen	Free thyroxine: 12 pmol/L	Hemoglobin A1c: 6.0%
Yeast: not seen	Free triiodothyronine: 3.4 pmol/L	

*HPF* high-power field, *CV* coefficient of variation, *SD* standard deviation

## Management

In our wound-care center, the patient's foot ulcer was initially examined using color flow Doppler and magnetic resonance imaging (MRI). No abnormalities were noted in the circulatory system and no evidence of osteomyelitis was found. Next, a wound swab sample was obtained from the ulcer under sterile conditions and was sent to the microbiology department of Imam Khomeini Hospital within 1 hour after sampling. The sample was then cultivated for aerobic and anaerobic organisms. The cultivation was conducted using MacConkey agar (MAC) to identify gram-positive and gram-negative bacteria. The culture media were kept for one day at 37 °C, and coagulase and catalase tests were also used to differentiate gram-positive bacteria. After identifying the type of bacteria, antibacterial susceptibility testing was conducted using the disk diffusion method, using Mueller–Hinton (MH) agar as the growth medium. Based on the laboratory findings, *Staphylococcus aureus* was revealed to be the cause of wound infection (Table 3).

**Table 3:** Results of patient's wound culture

Wound culture	Results
Pathogen	*Staphylococcus aureus*
Sensitive	Amikacin, vancomycin, nitrofurantoin
Resistant	Ofloxacin, trimethoprim, sulfamethoxazole
Intermediate	Clindamycin, erythromycin, ciprofloxacin
White blood cell count	4–6/HPF
Red blood cell count (direct smear)	1–2/HPF
Bacteria	Moderate

*HPF* high-power field

A blood culture test was obtained to detect foreign invaders including aerobes, anaerobes, and other microorganisms (two blood samples were taken from different veins). The result of blood culture was negative, and no microorganisms were detected.

**Fig. 4:**
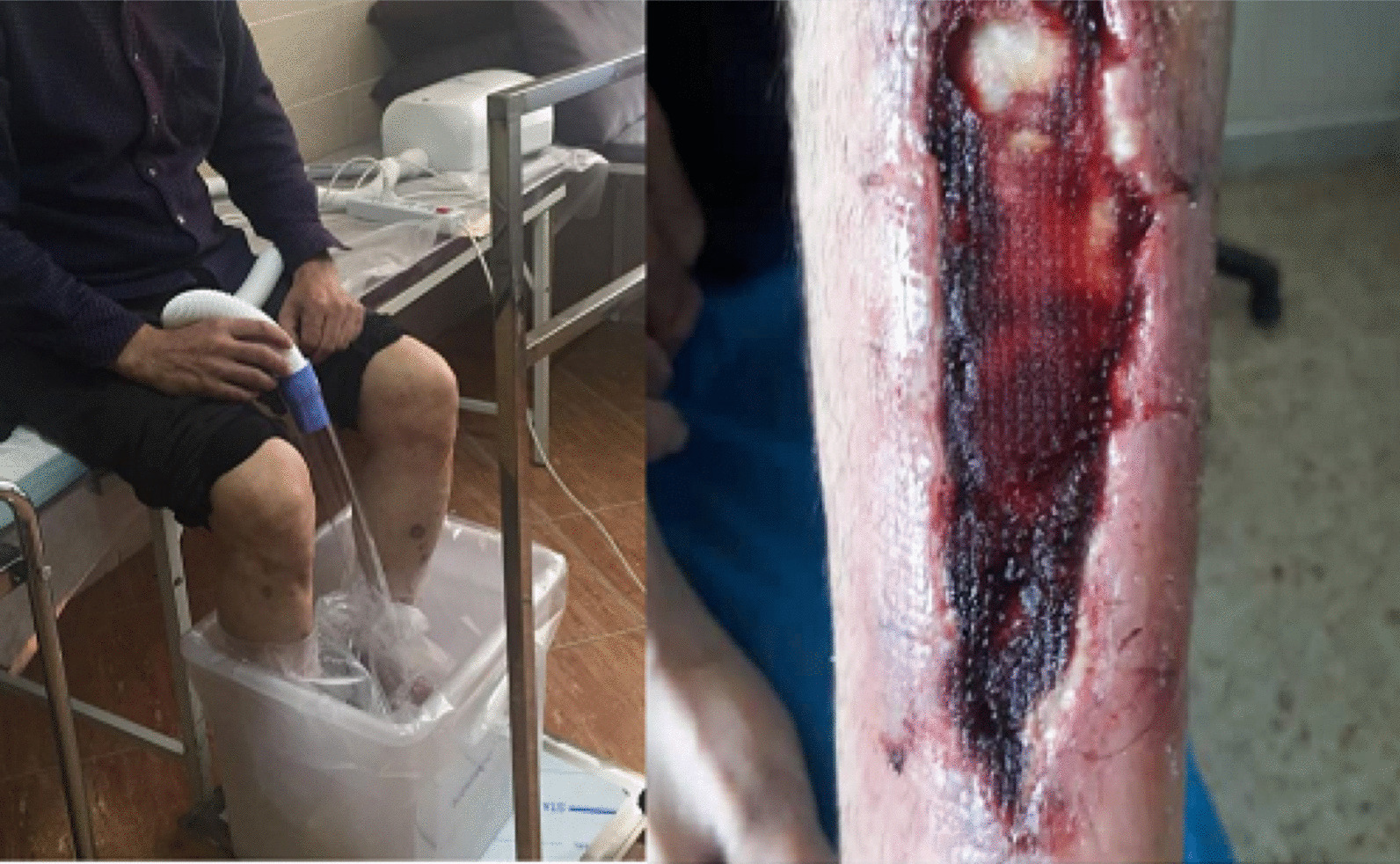
Silver dressing applied following ozone therapy

In the first step, the patient's necrotic tissues were irrigated and then debrided using mechanical debridement and saline. The patient then underwent a 70 μg/dL dose of ozone therapy over a 30-day period in 10 sessions (one 20-minute session every 3 days). The above process was conducted in an ozone-resistant plastic bag in order to trap ozone gas and create excessive exposure between the gas and the ulcer. The ozone was generated using a MOG003 ozone generator (Fig. 4).

**Fig. 5:**
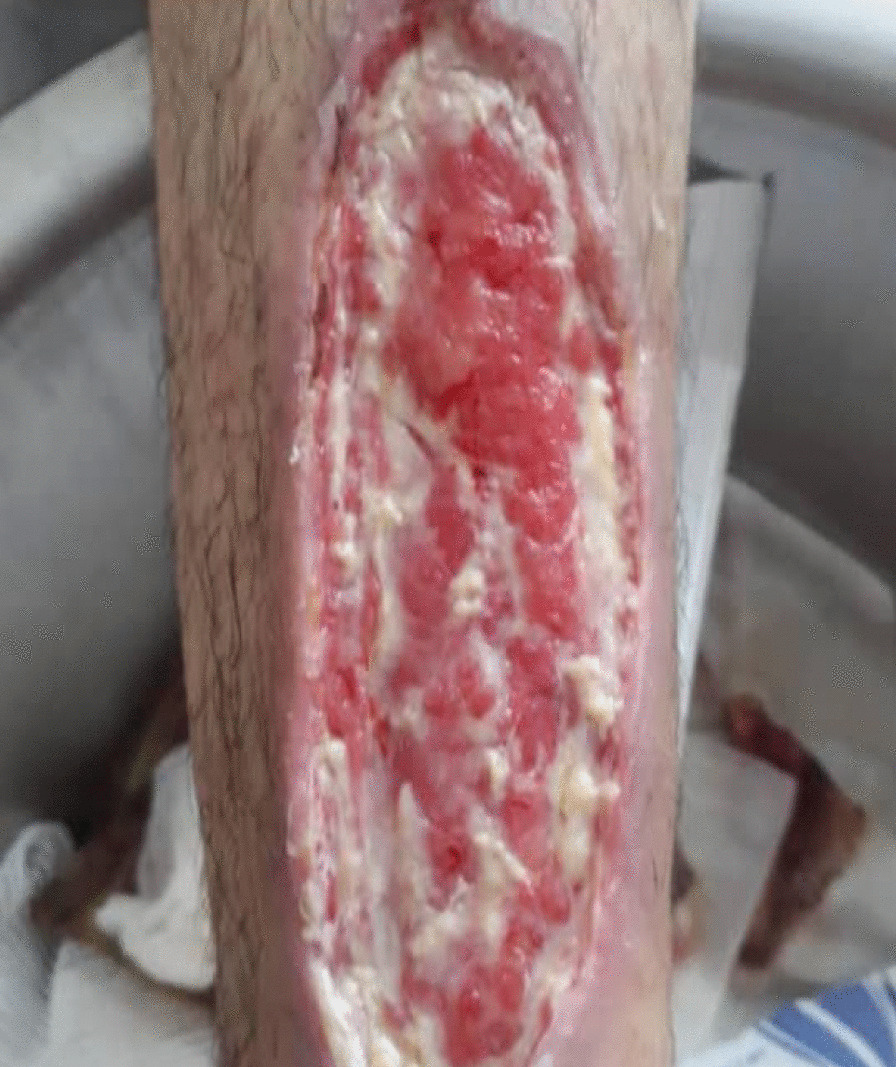
Diabetic foot ulcer after six sessions of ozone therapy

**Fig. 6:**
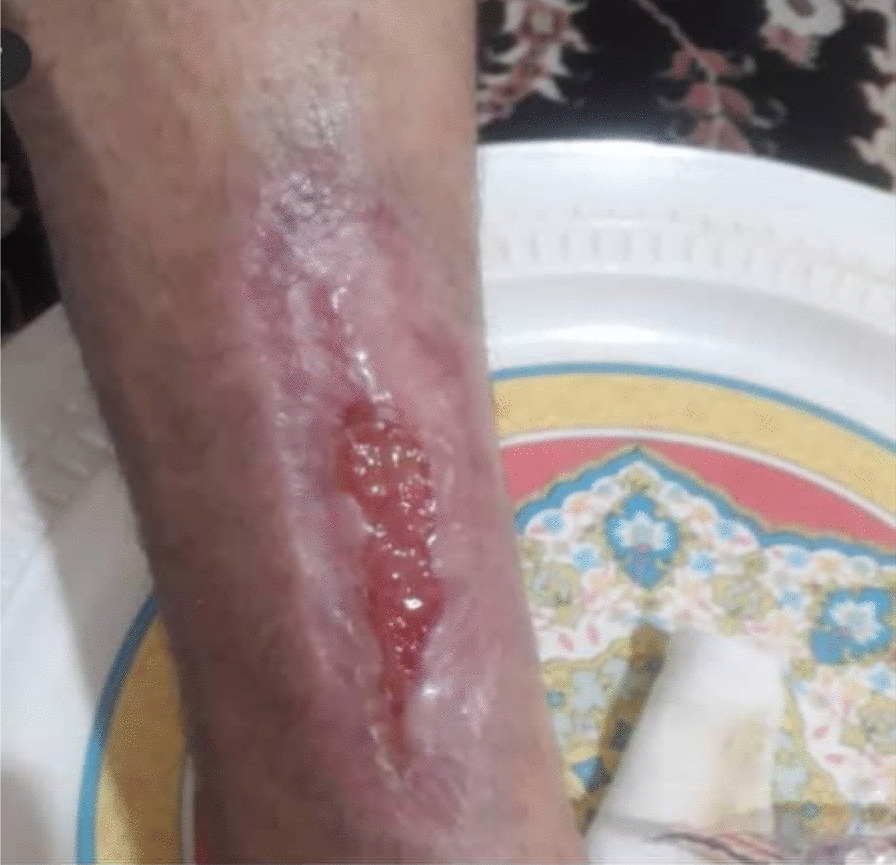
Diabetic foot ulcer of the patient after 1 month

Between each session, the patient's wound was wrapped in silver-containing gauze bandages (Fig. 4). After six sessions of ozone therapy, all the deep parts of the foot ulcer were filled due to the rapid growth of granulation tissue (Fig. 5). At this time, the patient was instructed to take his medication regularly and to adhere to a diabetic diet program. Additionally, he was instructed to avoid pressure on the repairing tissue throughout the treatment period. On 4 June 2020, about 1 month after the above treatment program, the patient's foot ulcer had healed completely, and he was discharged from our wound-care service in good and stable general condition (Fig. 6).

## Discussion

**Fig. 7:**
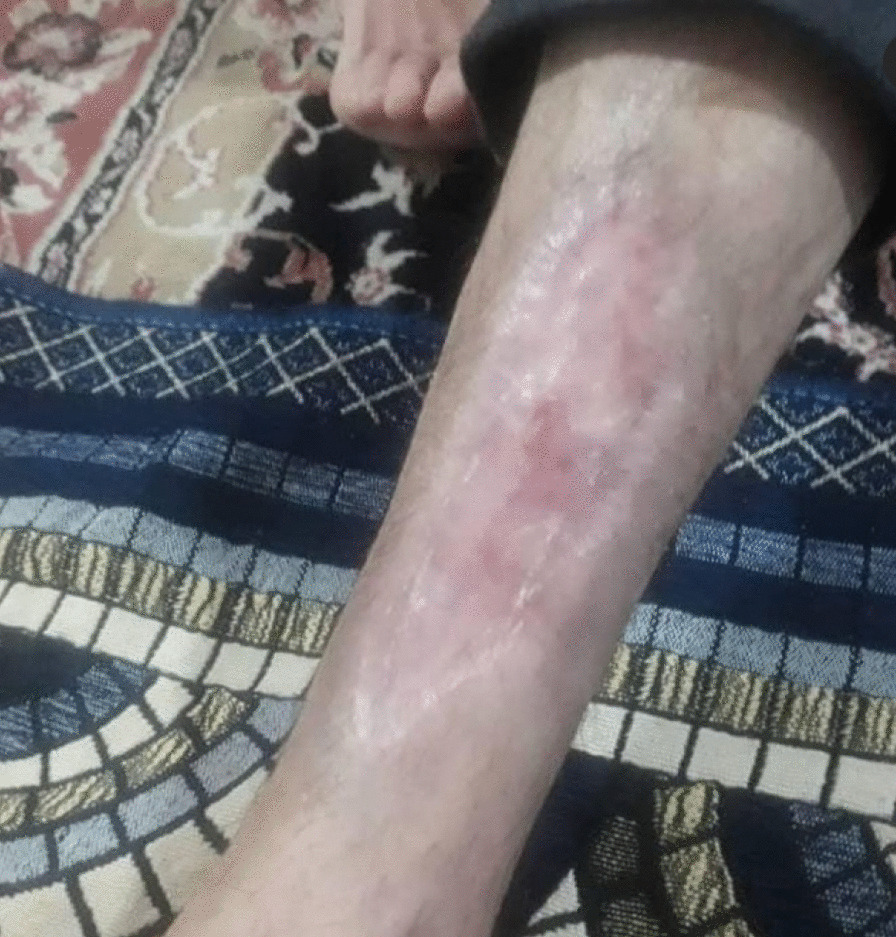
Diabetic foot ulcer of the patient after about 4 months

In this case report, the patient was injured in a bicycle accident. Because of his primary education level and lack of adherence to a proper diet, his blood sugar level was out of control. This condition resulted in an unhealed diabetic foot ulcer. First, he was hospitalized for a week in Imam Khomeini Hospital, and his wound was sutured (Fig. 2) and treated with intravenous administration of antibiotics along with saline wound dressing twice a day. The patient was discharged from the hospital with orally administered antibiotics, but his foot ulcer did not heal (Fig. 3), so he was referred to our wound-care team. Given the lack of underlying chronic respiratory or cardiovascular disease and the site of the DFU, which was an ideal wound for safe and side-effect-free ozone therapy, we commenced this method of wound treatment along with a silver dressing (which is unique in this case compared to other available literature). We used a 70 μg/dL dose of ozone therapy for a 30-day period that included 10 sessions of ozone therapy along with a silver dressing to treat the patient's DFU (Fig. 4). The patient was also instructed on diabetic diet and medications as well as diabetes self-care. After about 1 month, at the end of the treatment process, on 4 June 2020, the patient was discharged from our service in good general condition (Fig. 6). After about 4 months, the patient's DFU had healed completely (Fig. 7).

DFUs are a life-threatening and debilitating complication of advanced diabetes. Performing an amputation due to foot infection, necrosis, and osteomyelitis causes socio-psychological burden and lifestyle changes in these patients. Therefore, an appropriate therapeutic approach is very important for the management of DFUs [11]. Preventive strategies including patient education and regular foot assessments for PVD and neuropathy are the main components of DFU management [4]. Because DFUs are caused by multiple and complex pathological mechanisms, conventional treatment methods are associated with low success, and thus the treatment of these ulcers requires a new and innovative therapeutic approach [7]. In this case report, it was found that ozone therapy miraculously improved the healing of the patient's DFU. It has also been found that in addition to the antibacterial effects to prevent the progression of infection, ozone therapy releases growth factors that eventually heal tissue wounds [12].

Another mechanism of ozone therapy which has been observed in these patients is its positive effects on glucose metabolism. Ozone gas causes more glucose to enter the erythrocytes, which in turn causes hemoglobin to release more oxygen into the tissues and prevents tissue hypoxia, so it plays a key role in the emergence of DFUs [12, 13]. The essential key to success in treating DFUs is controlling the level of blood glucose at the optimal range to prevent microcirculation changes [14]. Regardless of the disadvantages of ozone therapy, including ozone gas toxicity, its clinical usefulness depends on the concentration, administration to the suitable site, and the type of treatment [10]. One of the major disadvantages of ozone therapy is its toxic effects on the respiratory tract. Other side effects include coughing, nausea, vomiting, and headache (in the case of entering the mouth, nose, or eyes) [11]. Patients who receive ozone therapy occasionally experience a Herxheimer reaction, which causes flu-like symptoms and other short-term side effects [15]. Ozone therapy is an alternative approach that utilizes ozone. This method is controversial because of the concerns around its efficiency and safety [10]. The US Food and Drug Administration (FDA) recently announced that ozone is a toxic gas and has no known effective applications in preventive medicine [16]. In Iran, the extensive use of ozone therapy as a treatment is prohibited, although its restricted application is authorized in studies. In this case, no side effects were reported during, immediately after, or 4 months following ozone therapy.

In line with the results of our study, Kadir *et al*. investigated the effect of ozone therapy on reducing bacterial colonization and healing of DFUs in patients with second- and third-degree DFUs. They showed that routine care combined with ozone therapy can have a significant effect on the healing of DFUs in these patients [8]. Rosul *et al*. showed that topical and systemic ozone therapy is effective in reducing wound size, reducing hospital stay, and producing more antioxidants in patients with DFUs [17]. Moreover, Wen *et al*. investigated the application of ozone therapy in DFUs and revealed that ozone therapy is potentially useful for closure of DFUs [18]. Zhang *et al*. reported that the efficacy rate, wound size reduction, and ulcer healing was remarkably higher at the end of treatment in the ozone therapy group compared to the control group [19]. Kushmakov *et al*. showed that the use of local ozone therapy can decrease the possibility of infection and treatment duration [20].

Izadi *et al*. examined the effect of ozone therapy in DFU healing on two groups. The control group received only routine treatment of DFUs and the intervention group received routine treatment along with ozone therapy twice a week. The results showed that ozone therapy was very effective in treating DFUs and reducing recovery time in the intervention group compared with the control group [2]. This result was consistent with the results of our study. Teuvov *et al*. (2017) also showed that ozone therapy as a complementary method reduced the length of hospitalization in patients with DFU and accelerated their recovery [12]. In a case report by Aytacoglu *et al*., the use of ozone therapy was reported to prevent foot amputation in a 67-year-old woman with a DFU [21]. The above evidence of the positive effect of ozone therapy may indicate a novel horizon for treatment of patients with DFUs, and we cannot simply abandon this alternative approach.

## Conclusion

Treatment of DFUs is very important, and there are still many concerns in this area. Thus, the use of alternative or complementary strategies is needed. Ozone therapy is less invasive than other alternative treatments for DFU such as maggot therapy (MT) or negative-pressure wound therapy (NPWT), which makes it easier for patients to accept it. This case report confirms the efficiency of ozone therapy combined with silver dressing as an adjunct to DFU treatment. Therefore, it is suggested that this method should be widely used to accelerate the recovery period throughout the treatment of DFU due to its efficacy and cost-effectiveness.

## Data Availability

Not applicable.

## Abbreviations

DFU: Diabetic foot ulcer
PVD: Peripheral vascular disease
TURP: Transurethral resection of the prostate
MRI: Magnetic resonance imaging
MAC: MacConkey agar
FDA: Food and Drug Administration
MT: Maggot therapy
NPWT: Negative-pressure wound therapy
